# 5-Chloro-3-ethyl­sulfinyl-7-methyl-2-(4-methyl­phen­yl)-1-benzo­furan

**DOI:** 10.1107/S1600536814005601

**Published:** 2014-03-19

**Authors:** Hong Dae Choi, Pil Ja Seo, Uk Lee

**Affiliations:** a Department of Chemistry, Dongeui University, San 24 Kaya-dong, Busanjin-gu, Busan 614-714, Republic of Korea; b Department of Chemistry, Pukyong National University, 599-1 Daeyeon 3-dong, Nam-gu, Busan 608-737, Republic of Korea

## Abstract

In the title compound, C_18_H_17_ClO_2_S, the dihedral angle between the mean planes of the benzo­furan ring system and the methyl­phenyl ring is 14.50 (4)°. The centroid–centroid distances between the benzene and the methyl­phenyl rings are 3.827 (2) and 3.741 (2) Å, while the centroid–centroid distance between the furan and methyl­phenyl rings is 3.843 (2) Å. These distances indicate π–π inter­actions; on the other hand, the inter­planar angles between the benzene and methyl­phenyl rings, and between the furan and methyl­phenyl rings are 13.89 (4) and 15.53 (4)°, respectively. In the crystal, the mol­ecules stack along the *a*-axis direction.

## Related literature   

For background information about related compounds and their crystal structures, see Choi *et al.* (2010*a* ▶,*b* ▶). For π–π stacking in metal complexes with aromatic nitro­gen ligands, see: Janiak (2000 ▶).
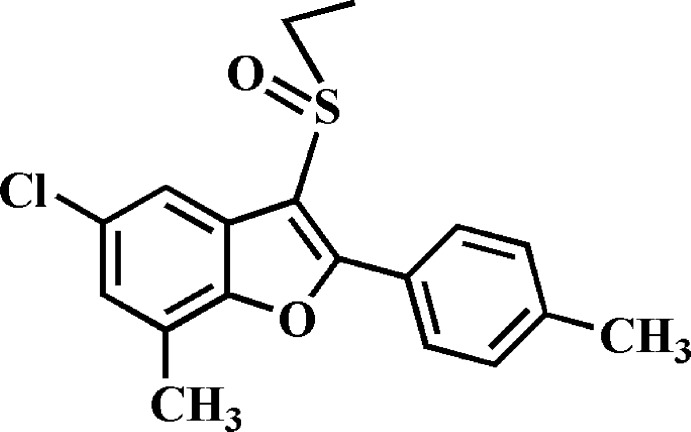


## Experimental   

### 

#### Crystal data  

C_18_H_17_ClO_2_S
*M* *_r_* = 332.83
Triclinic, 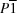

*a* = 7.3638 (5) Å
*b* = 10.2524 (6) Å
*c* = 11.8335 (7) Å
α = 68.949 (3)°
β = 89.362 (3)°
γ = 71.460 (3)°
*V* = 785.11 (8) Å^3^
*Z* = 2
Mo *K*α radiation
μ = 0.38 mm^−1^
*T* = 173 K
0.46 × 0.37 × 0.33 mm

#### Data collection  

Bruker SMART APEXII CCD diffractometer
Absorption correction: multi-scan (*SADABS*; Bruker, 2009 ▶) *T*_min_ = 0.691, *T*_max_ = 0.746
13937 measured reflections
3759 independent reflections
3348 reflections with *I* > 2σ(*I*)
*R*_int_ = 0.031

#### Refinement  

*R*[*F* ^2^ > 2σ(*F* ^2^)] = 0.034
*wR*(*F* ^2^) = 0.100
*S* = 1.06
3759 reflections
203 parameters
H-atom parameters constrained
Δρ_max_ = 0.31 e Å^−3^
Δρ_min_ = −0.29 e Å^−3^

### 

Data collection: *APEX2* (Bruker, 2009 ▶); cell refinement: *SAINT* (Bruker, 2009 ▶); data reduction: *SAINT*; program(s) used to solve structure: *SHELXS97* (Sheldrick, 2008 ▶); program(s) used to refine structure: *SHELXL97* (Sheldrick, 2008 ▶); molecular graphics: *ORTEP-3 for Windows* (Farrugia, 2012 ▶) and *DIAMOND* (Brandenburg, 1998 ▶); software used to prepare material for publication: *SHELXL97*.

## Supplementary Material

Crystal structure: contains datablock(s) I. DOI: 10.1107/S1600536814005601/fb2296sup1.cif

Structure factors: contains datablock(s) I. DOI: 10.1107/S1600536814005601/fb2296Isup2.hkl

Supporting information file. DOI: 10.1107/S1600536814005601/fb2296Isup3.cml

CCDC reference: 991267

Additional supporting information:  crystallographic information; 3D view; checkCIF report

## Figures and Tables

**Fig. 1.**
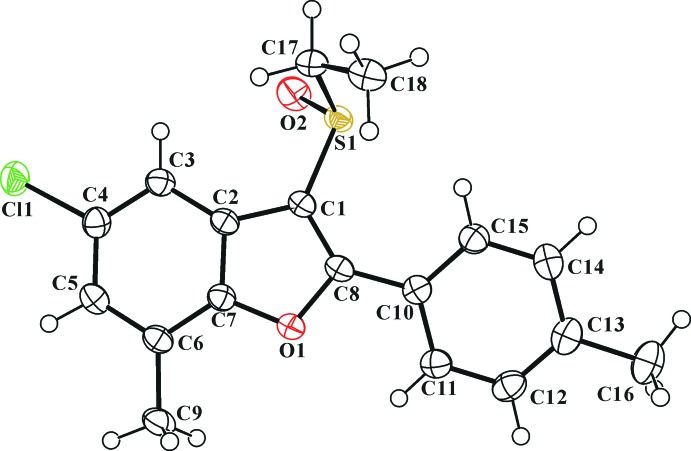
The title molecule with the atom numbering scheme. The displacement ellipsoids are drawn at the 50% probability level. The hydrogen atoms are presented as small spheres of arbitrary radius.

**Fig. 2.**
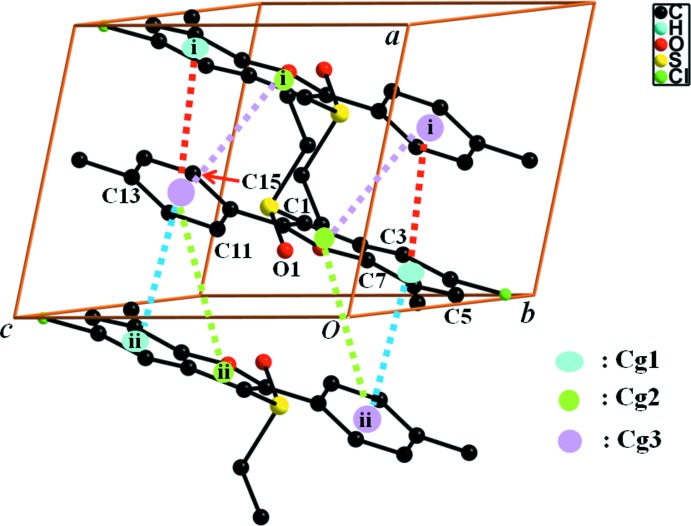
A view of possible π···π interactions (dotted lines) in the crystal structure of the title compound. The H-atoms have been omitted for clarity. [Symmetry codes: (i) -x + 1, -y + 1, -z + 1; (ii) -x, -y + 1, -z + 1.]

## supplementary crystallographic information

### 1. Comment

As a part of our ongoing study of 5-chloro-3-ethylsulfinyl-7-methyl-1-benzofuran derivatives which contain 4-fluorophenyl and 4 iodophenyl substituents in the 2-position (Choi *et al.* (2010*a*,*b*) for the F and I-compound, respectively), we report the crystal structure of the title compound.

In the title molecule (Fig. 1), the benzofuran unit is essentially planar, with the mean deviation from the least-squares plane defined by the nine constituent atoms which equals to 0.016 (1) Å. The 4-methylphenyl ring is also essentially planar, with the mean deviation of 0.004 (1) Å from the least-squares plane defined by the six core-ring atoms. The dihedral angle between the benzofuran ring system and the core of the 4-methylphenyl rings is 14.50 (4)°.

Let the centroid names *Cg*1, *Cg*2 and *Cg*3 be assigned to the benzene ring (C2–C7), the furan ring (C1/C2/C7/O1/C8) and the core of 4-methylphenyl ring (C10–C15), respectively: The centroid–centroid separations of *Cg*1···*Cg*3^i^, *Cg*1···*Cg*3^ii^ and Cg2···Cg3^i^ are 3.827 (2), 3.741 (2) and 3.843 (2) Å, respectively. (The symmetry codes are: (i) -*x* + 1, -*y* + 1, -*z* + 1; (ii) -*x*, -*y* + 1, -*z* + 1.)

The interplanar angles between the benzene and the core of 4-methylphenyl ring and between the furan and the core of 4-methylphenyl ring equal to 13.89 (4) and 15.53 (4)°, respectively. These angles are quite large for the rings being in π-electron···π-electron interactions as it follows from the study by Janiak (2000) who investigated π–π stacking in metal complexes with aromatic nitrogen ligands. According to Fig. 8 of Janiak's study, the interplanar angles between the rings that are involved in π-electron···π-electron interactions are less than 10° in overwhelming majority of cases.

### 2. Experimental

3-Chloroperoxybenzoic acid (77%, 224 mg, 1.0 mmol) was added in small portions to the stirred solution of 5-chloro-3-ethylsulfanyl-7-methyl-2-(4-methylphenyl)-1-benzofuran (285 mg, 0.9 mmol) in dichloromethane (30 ml) at 273 K. The mixture was washed with saturated sodium hydrogen carbonate solution after having been stirred at room temperature for 4h. The organic layer was separated, dried over magnesium sulfate, filtered and concentrated at reduced pressure.

The residue was purified by column chromatography (hexane–ethyl acetate, 1:1*v*/*v*) to afford the title compound as a colourless solid [yield 71%, m.p. 392–393 K; *R*_f_ = 0.56 (hexane–ethyl acetate, 1:1 *v*/*v*)]. Single crystals suitable for X-ray diffraction were prepared by slow evaporation of the 5% solution of the title compound in acetone at room temperature. The average crystal size was approximately 1.1 × 1.3 × 0.8 mm. (The measured crystal was cut from a large one.) The crystals are soluble in polar solvents.

### 3. Refinement

All the hydrogen atoms were observed in the difference electron density map. However, they were situated into the idealized positions and refined using a riding-model approximation. The used constraints: C—H = 0.95 Å for aryl, 0.98 Å for methyl and for 0.99 Å for methylene H atoms. *U*_iso_(H) = 1.2*U*_eq_(C) for aryl and methylene, while 1.5*U*_eq_(C) for the methyl H atoms. The positions of methyl hydrogens were optimized using the *SHELXL*-97's command AFIX 137 (Sheldrick, 2008).

### Crystal data

**Table:**

C_18_H_17_ClO_2_S	*Z* = 2
*M**_r_* = 332.83	*F*(000) = 348
Triclinic, *P*1	*D*_x_ = 1.408 Mg m^−^^3^
Hall symbol: -P 1	Melting point = 392–393 K
*a* = 7.3638 (5) Å	Mo *K*α radiation, λ = 0.71073 Å
*b* = 10.2524 (6) Å	Cell parameters from 8286 reflections
*c* = 11.8335 (7) Å	θ = 2.3–28.4°
α = 68.949 (3)°	µ = 0.38 mm^−^^1^
β = 89.362 (3)°	*T* = 173 K
γ = 71.460 (3)°	Block, colourless
*V* = 785.11 (8) Å^3^	0.46 × 0.37 × 0.33 mm

### Data collection

**Table:**

Bruker SMART APEXII CCD diffractometer	3759 independent reflections
Radiation source: rotating anode	3348 reflections with *I* > 2σ(*I*)
Graphite multilayer monochromator	*R*_int_ = 0.031
Detector resolution: 10.0 pixels mm^-1^	θ_max_ = 28.0°, θ_min_ = 2.3°
φ and ω scans	*h* = −9→9
Absorption correction: multi-scan (*SADABS*; Bruker, 2009)	*k* = −13→13
*T*_min_ = 0.691, *T*_max_ = 0.746	*l* = −15→15
13937 measured reflections	

### Refinement

**Table:**

Refinement on *F*^2^	Secondary atom site location: difference Fourier map
Least-squares matrix: full	Hydrogen site location: difference Fourier map
*R*[*F*^2^ > 2σ(*F*^2^)] = 0.034	H-atom parameters constrained
*wR*(*F*^2^) = 0.100	*w* = 1/[σ^2^(*F*_o_^2^) + (0.0517*P*)^2^ + 0.2412*P*] where *P* = (*F*_o_^2^ + 2*F*_c_^2^)/3
*S* = 1.06	(Δ/σ)_max_ < 0.001
3759 reflections	Δρ_max_ = 0.31 e Å^−^^3^
203 parameters	Δρ_min_ = −0.29 e Å^−^^3^
0 restraints	Extinction correction: *SHELXL97* (Sheldrick, 2008), Fc^*^=kFc[1+0.001xFc^2^λ^3^/sin(2θ)]^-1/4^
Primary atom site location: structure-invariant direct methods	Extinction coefficient: 0.006 (2)

### Special details

**Table:**

Geometry. All esds (except the esd in the dihedral angle between two l.s. planes) are estimated using the full covariance matrix. The cell esds are taken into account individually in the estimation of esds in distances, angles and torsion angles; correlations between esds in cell parameters are only used when they are defined by crystal symmetry. An approximate (isotropic) treatment of cell esds is used for estimating esds involving l.s. planes.
Refinement. Refinement of F^2^ against ALL reflections. The weighted R-factor wR and goodness of fit S are based on F^2^, conventional R-factors R are based on F, with F set to zero for negative F^2^. The threshold expression of F^2^ > 2sigma(F^2^) is used only for calculating R-factors(gt) etc. and is not relevant to the choice of reflections for refinement. R-factors based on F^2^ are statistically about twice as large as those based on F, and R- factors based on ALL data will be even larger.

### Fractional atomic coordinates and isotropic or equivalent isotropic displacement parameters (Å^2^)

**Table:**

	*x*	*y*	*z*	*U*_iso_*/*U*_eq_	
Cl1	0.02297 (6)	0.70926 (4)	−0.07104 (3)	0.03932 (13)	
S1	0.36271 (5)	0.19161 (4)	0.40911 (3)	0.02784 (11)	
O1	0.19351 (14)	0.58273 (10)	0.44275 (8)	0.0265 (2)	
O2	0.21419 (17)	0.18248 (12)	0.32973 (11)	0.0411 (3)	
C1	0.29375 (19)	0.37881 (14)	0.39556 (12)	0.0251 (3)	
C2	0.20899 (19)	0.50599 (14)	0.28375 (12)	0.0248 (3)	
C3	0.1735 (2)	0.52797 (15)	0.16126 (13)	0.0279 (3)	
H3	0.2128	0.4481	0.1335	0.033*	
C4	0.0781 (2)	0.67216 (16)	0.08294 (13)	0.0290 (3)	
C5	0.0153 (2)	0.79189 (15)	0.12135 (13)	0.0298 (3)	
H5	−0.0515	0.8884	0.0634	0.036*	
C7	0.14795 (19)	0.62695 (15)	0.31933 (12)	0.0251 (3)	
C6	0.0489 (2)	0.77201 (15)	0.24256 (13)	0.0273 (3)	
C8	0.28210 (19)	0.43087 (14)	0.48790 (12)	0.0249 (3)	
C9	−0.0186 (2)	0.89610 (16)	0.28858 (15)	0.0343 (3)	
H9A	−0.1057	0.9849	0.2243	0.052*	
H9B	0.0929	0.9177	0.3116	0.052*	
H9C	−0.0870	0.8668	0.3599	0.052*	
C10	0.3426 (2)	0.36521 (15)	0.61893 (12)	0.0262 (3)	
C11	0.2685 (2)	0.44896 (16)	0.69082 (13)	0.0299 (3)	
H11	0.1763	0.5457	0.6543	0.036*	
C12	0.3291 (2)	0.39130 (18)	0.81450 (14)	0.0343 (3)	
H12	0.2787	0.4499	0.8615	0.041*	
C13	0.4619 (2)	0.24959 (18)	0.87129 (13)	0.0339 (3)	
C14	0.5333 (2)	0.16600 (17)	0.80041 (14)	0.0338 (3)	
H14	0.6229	0.0683	0.8379	0.041*	
C15	0.4761 (2)	0.22262 (16)	0.67607 (13)	0.0297 (3)	
H15	0.5282	0.1639	0.6294	0.036*	
C16	0.5257 (3)	0.1898 (2)	1.00639 (14)	0.0458 (4)	
H16A	0.6069	0.0852	1.0331	0.069*	
H16B	0.4121	0.1986	1.0509	0.069*	
H16C	0.5994	0.2468	1.0229	0.069*	
C17	0.5744 (2)	0.18473 (17)	0.32871 (14)	0.0336 (3)	
H17A	0.6062	0.1004	0.3012	0.040*	
H17B	0.5467	0.2772	0.2556	0.040*	
C18	0.7460 (2)	0.16692 (17)	0.40993 (15)	0.0363 (3)	
H18A	0.7127	0.2480	0.4401	0.054*	
H18B	0.8561	0.1696	0.3630	0.054*	
H18C	0.7798	0.0717	0.4791	0.054*	

### Atomic displacement parameters (Å^2^)

**Table:**

	*U*^11^	*U*^22^	*U*^33^	*U*^12^	*U*^13^	*U*^23^
Cl1	0.0511 (3)	0.0362 (2)	0.02820 (19)	−0.01466 (18)	−0.00402 (16)	−0.00909 (15)
S1	0.0307 (2)	0.02136 (17)	0.03107 (19)	−0.00773 (14)	0.00090 (14)	−0.01020 (13)
O1	0.0277 (5)	0.0223 (5)	0.0278 (5)	−0.0054 (4)	0.0023 (4)	−0.0101 (4)
O2	0.0422 (7)	0.0359 (6)	0.0484 (7)	−0.0158 (5)	−0.0071 (5)	−0.0166 (5)
C1	0.0235 (6)	0.0218 (6)	0.0280 (6)	−0.0057 (5)	0.0031 (5)	−0.0089 (5)
C2	0.0210 (6)	0.0230 (6)	0.0296 (7)	−0.0065 (5)	0.0029 (5)	−0.0098 (5)
C3	0.0274 (7)	0.0263 (6)	0.0304 (7)	−0.0080 (5)	0.0016 (5)	−0.0119 (5)
C4	0.0283 (7)	0.0304 (7)	0.0275 (7)	−0.0106 (6)	−0.0006 (5)	−0.0092 (5)
C5	0.0274 (7)	0.0240 (6)	0.0334 (7)	−0.0067 (5)	−0.0010 (6)	−0.0070 (5)
C7	0.0225 (6)	0.0241 (6)	0.0280 (6)	−0.0068 (5)	0.0017 (5)	−0.0100 (5)
C6	0.0226 (6)	0.0236 (6)	0.0338 (7)	−0.0059 (5)	0.0019 (5)	−0.0103 (5)
C8	0.0222 (6)	0.0216 (6)	0.0304 (7)	−0.0068 (5)	0.0041 (5)	−0.0098 (5)
C9	0.0339 (8)	0.0237 (7)	0.0410 (8)	−0.0028 (6)	0.0020 (6)	−0.0130 (6)
C10	0.0258 (7)	0.0275 (6)	0.0275 (6)	−0.0121 (5)	0.0056 (5)	−0.0102 (5)
C11	0.0320 (7)	0.0292 (7)	0.0318 (7)	−0.0132 (6)	0.0080 (6)	−0.0129 (6)
C12	0.0398 (8)	0.0396 (8)	0.0318 (7)	−0.0199 (7)	0.0115 (6)	−0.0173 (6)
C13	0.0327 (8)	0.0441 (8)	0.0278 (7)	−0.0205 (7)	0.0064 (6)	−0.0104 (6)
C14	0.0294 (7)	0.0340 (7)	0.0315 (7)	−0.0097 (6)	0.0021 (6)	−0.0058 (6)
C15	0.0270 (7)	0.0313 (7)	0.0304 (7)	−0.0088 (6)	0.0042 (5)	−0.0121 (6)
C16	0.0473 (10)	0.0616 (11)	0.0283 (8)	−0.0238 (9)	0.0054 (7)	−0.0117 (7)
C17	0.0341 (8)	0.0309 (7)	0.0331 (7)	−0.0029 (6)	0.0056 (6)	−0.0160 (6)
C18	0.0332 (8)	0.0325 (7)	0.0451 (9)	−0.0107 (6)	0.0082 (6)	−0.0172 (7)

### Geometric parameters (Å, º)

**Table:**

Cl1—C4	1.7465 (15)	C10—C15	1.397 (2)
S1—O2	1.4962 (12)	C10—C11	1.4054 (19)
S1—C1	1.7686 (13)	C11—C12	1.384 (2)
S1—C17	1.8129 (16)	C11—H11	0.9500
O1—C7	1.3746 (16)	C12—C13	1.389 (2)
O1—C8	1.3782 (15)	C12—H12	0.9500
C1—C8	1.3689 (19)	C13—C14	1.391 (2)
C1—C2	1.4498 (18)	C13—C16	1.510 (2)
C2—C7	1.3905 (18)	C14—C15	1.387 (2)
C2—C3	1.3988 (19)	C14—H14	0.9500
C3—C4	1.3811 (19)	C15—H15	0.9500
C3—H3	0.9500	C16—H16A	0.9800
C4—C5	1.401 (2)	C16—H16B	0.9800
C5—C6	1.388 (2)	C16—H16C	0.9800
C5—H5	0.9500	C17—C18	1.519 (2)
C7—C6	1.3868 (18)	C17—H17A	0.9900
C6—C9	1.5006 (19)	C17—H17B	0.9900
C8—C10	1.4597 (19)	C18—H18A	0.9800
C9—H9A	0.9800	C18—H18B	0.9800
C9—H9B	0.9800	C18—H18C	0.9800
C9—H9C	0.9800		
			
O2—S1—C1	106.72 (6)	C15—C10—C8	122.50 (13)
O2—S1—C17	106.62 (7)	C11—C10—C8	119.29 (12)
C1—S1—C17	98.27 (7)	C12—C11—C10	120.40 (14)
C7—O1—C8	107.00 (10)	C12—C11—H11	119.8
C8—C1—C2	107.17 (12)	C10—C11—H11	119.8
C8—C1—S1	127.41 (10)	C11—C12—C13	121.40 (14)
C2—C1—S1	124.75 (10)	C11—C12—H12	119.3
C7—C2—C3	119.38 (12)	C13—C12—H12	119.3
C7—C2—C1	104.93 (12)	C12—C13—C14	118.17 (14)
C3—C2—C1	135.63 (13)	C12—C13—C16	120.22 (15)
C4—C3—C2	116.30 (13)	C14—C13—C16	121.61 (15)
C4—C3—H3	121.8	C15—C14—C13	121.24 (14)
C2—C3—H3	121.8	C15—C14—H14	119.4
C3—C4—C5	123.25 (13)	C13—C14—H14	119.4
C3—C4—Cl1	119.41 (11)	C14—C15—C10	120.58 (14)
C5—C4—Cl1	117.29 (11)	C14—C15—H15	119.7
C6—C5—C4	121.19 (13)	C10—C15—H15	119.7
C6—C5—H5	119.4	C13—C16—H16A	109.5
C4—C5—H5	119.4	C13—C16—H16B	109.5
O1—C7—C6	124.11 (12)	H16A—C16—H16B	109.5
O1—C7—C2	110.69 (11)	C13—C16—H16C	109.5
C6—C7—C2	125.17 (13)	H16A—C16—H16C	109.5
C7—C6—C5	114.68 (12)	H16B—C16—H16C	109.5
C7—C6—C9	122.11 (13)	C18—C17—S1	111.08 (10)
C5—C6—C9	123.20 (13)	C18—C17—H17A	109.4
C1—C8—O1	110.19 (12)	S1—C17—H17A	109.4
C1—C8—C10	135.60 (12)	C18—C17—H17B	109.4
O1—C8—C10	114.20 (11)	S1—C17—H17B	109.4
C6—C9—H9A	109.5	H17A—C17—H17B	108.0
C6—C9—H9B	109.5	C17—C18—H18A	109.5
H9A—C9—H9B	109.5	C17—C18—H18B	109.5
C6—C9—H9C	109.5	H18A—C18—H18B	109.5
H9A—C9—H9C	109.5	C17—C18—H18C	109.5
H9B—C9—H9C	109.5	H18A—C18—H18C	109.5
C15—C10—C11	118.19 (13)	H18B—C18—H18C	109.5
			
O2—S1—C1—C8	131.72 (13)	C4—C5—C6—C7	0.2 (2)
C17—S1—C1—C8	−118.07 (13)	C4—C5—C6—C9	−178.83 (14)
O2—S1—C1—C2	−37.66 (13)	C2—C1—C8—O1	0.68 (15)
C17—S1—C1—C2	72.54 (13)	S1—C1—C8—O1	−170.20 (10)
C8—C1—C2—C7	−1.27 (15)	C2—C1—C8—C10	−178.15 (15)
S1—C1—C2—C7	169.92 (10)	S1—C1—C8—C10	11.0 (2)
C8—C1—C2—C3	−178.44 (15)	C7—O1—C8—C1	0.20 (14)
S1—C1—C2—C3	−7.3 (2)	C7—O1—C8—C10	179.30 (11)
C7—C2—C3—C4	0.1 (2)	C1—C8—C10—C15	15.7 (2)
C1—C2—C3—C4	176.96 (15)	O1—C8—C10—C15	−163.06 (12)
C2—C3—C4—C5	−1.2 (2)	C1—C8—C10—C11	−165.94 (15)
C2—C3—C4—Cl1	−178.57 (10)	O1—C8—C10—C11	15.27 (18)
C3—C4—C5—C6	1.0 (2)	C15—C10—C11—C12	0.8 (2)
Cl1—C4—C5—C6	178.51 (11)	C8—C10—C11—C12	−177.59 (13)
C8—O1—C7—C6	176.97 (13)	C10—C11—C12—C13	−0.8 (2)
C8—O1—C7—C2	−1.06 (14)	C11—C12—C13—C14	0.0 (2)
C3—C2—C7—O1	179.17 (11)	C11—C12—C13—C16	179.84 (14)
C1—C2—C7—O1	1.44 (15)	C12—C13—C14—C15	0.9 (2)
C3—C2—C7—C6	1.2 (2)	C16—C13—C14—C15	−179.02 (14)
C1—C2—C7—C6	−176.57 (13)	C13—C14—C15—C10	−0.8 (2)
O1—C7—C6—C5	−179.01 (12)	C11—C10—C15—C14	0.0 (2)
C2—C7—C6—C5	−1.3 (2)	C8—C10—C15—C14	178.34 (13)
O1—C7—C6—C9	0.0 (2)	O2—S1—C17—C18	−170.38 (10)
C2—C7—C6—C9	177.75 (13)	C1—S1—C17—C18	79.34 (11)
